# Inhibition of Inflammatory Signals “HMGB1, NLRP3, TNF-α, IL-1β” and Oxidative Stress in Diclofenac-Induced Hepatotoxicity in Rats Using Hydroalcoholic Extract of *Dracocephalum kotschyi*

**DOI:** 10.5812/ijpr-162656

**Published:** 2025-08-11

**Authors:** Maryam Karimi-Dehkordi, Firoozeh Saghaei, Erfan Hoseini

**Affiliations:** 1 Department of Veterinary, Shk.C., Islamic Azad University, Shahrekord, Iran

**Keywords:** *Dracocephalum kotschyi*, Diclofenac, Oxidative Stress, Tumor Necrosis Factor-alpha, Interleukin-1 Beta, HMGB1 Protein, NLR Proteins

## Abstract

**Background:**

Excessive and prolonged use of nonsteroidal anti-inflammatory drugs (NSAIDs) can cause hepatotoxicity. However, prevention and treatment of this complication remain challenging.

**Objectives:**

This study investigated the potential of the hydroalcoholic extract of *Dracocephalum kotschyi* (HEDK) in inhibiting diclofenac (DIC)-induced hepatotoxicity in rats.

**Methods:**

Forty-two male Wistar rats were divided into six groups: Control group, DIC group, DIC+HEDK group in three different doses, and DIC+silymarin (SLY) group. The rats were treated for 7 days. Then, by inducing anesthesia, collecting blood from the heart, and isolating the liver, the effects of HEDK were evaluated by measuring the liver enzymes, antioxidant enzymes (using the calorimetric method), and inflammatory factors (using the RT-PCR method). Histopathological changes of the liver were also studied.

**Results:**

The DIC significantly increased the levels of enzymes in the liver, such as ALT, AST, and ALP, lipid oxidation product, malondialdehyde (MDA), and cytokines, interleukin 1 beta (IL-1β), tumor necrosis factor α (TNF-α), NOD-like receptor protein 3 (NLRP3), and high-mobility group box 1 (HMGB1). It also decreased the levels of oxidative stress factors, superoxide dismutase (SOD), catalase (CAT), and glutathione peroxidase (GPx). Treatment with HEDK improved the liver’s biochemical parameters and significantly reduced the inflammatory cytokines. With the reduction of MDA, antioxidant enzymes also increased. The liver’s histological study showed the ameliorating effects of HEDK.

**Conclusions:**

The HEDK provided significant protection against DIC-induced toxicity due to its antioxidant effects and inhibition of inflammatory factors.

## 1. Background

Nonsteroidal anti-inflammatory drugs (NSAIDs) are used to reduce inflammation, pain, and fever. These drugs usually have few side effects at therapeutic doses, but administering high doses can cause toxic effects on the liver, kidneys, and stomach (1-3). Diclofenac (DIC), a drug in this class, is used to control mild to moderate pain and treat some inflammatory diseases (4). Repeated use of DIC has been shown to result in drug-induced liver injury (IDILI) (5). The production of quinone metabolites following the oxidative metabolism of DIC in the liver leads to the induction of oxidative stress and apoptosis in human and mouse hepatocytes in vitro (6). These effects are associated with mitochondrial dysfunction and tumor necrosis factor α (TNF-α)-induced inhibition of nuclear factor kappa B (NF-kB) (5). Immune reactions and hypersensitivity also play an important role in DIC-induced liver injury (6). Previous studies have shown that after the induction of liver injury by the use of DIC, some proinflammatory cytokines, such as interleukin 1 beta (IL-1β), TNF-α, and immune factors, are released into the blood (5). Therefore, the protection of the liver against DIC-induced toxicity seems essential. This protection can be achieved through the use of medicinal plant extracts, as their ability in liver disorder treatments has been demonstrated in the ever-expanding research that is being conducted.

Silymarin (SLY), a polyphenolic flavonoid extracted from *Silybum marianum*, is a type of medicinal plant extract that has been used in the treatment of some liver disorders, such as cirrhosis and chronic hepatitis, as well as hepatotoxicity caused by chemicals. The SLY has antioxidant and anti-inflammatory properties (7, 8), and in the present study, SLY was used as a standard drug. *Dracocephalum kotschyi*, a medicinal plant used in this study, is a member of the Lamiaceae family and contains compounds such as citral, limonene, luteolin, terpineol, apigenin, and other flavonoids. It is used in traditional medicine to treat some gastrointestinal and renal problems. Luteolin has anti-inflammatory, antioxidant, and antitumor properties and can be used to prevent cancer and some inflammatory disorders (9). The hydroalcoholic extract of *Dracocephalum kotschyi* (HEDK) inhibits the secretion of TNF-α and IL-1β from macrophages, indicating its capacity to reduce immune and inflammatory responses (10). In another study, HEDK was investigated in surgically induced intra-abdominal adhesions in rats, and its anti-inflammatory effect was observed (11). The apigenin extracted from *D. kotschyi* reduced the inflammation of acetic acid-induced colitis in mice (12).

## 2. Objectives

Given the widespread use of anti-inflammatory drugs and the possibility of their liver side effects, along with limited treatment options available, the present study aimed to investigate the anti-inflammatory effects of HEDK on DIC-induced hepatotoxicity in rats.

## 3. Methods

### 3.1. Drugs and Chemicals

The DIC was purchased from Caspian Tamin (Iran), and SLY was purchased from Jahan Shimi (Iran).

### 3.2. Preparation of Hydroalcoholic Extract of Dracocephalum kotschyi

The aerial part of *D. kotschyi* was collected from its natural habitat in Chaharmahal and Bakhtiari province and confirmed with Herbarium No. 654-D at the Medicinal Plants Research Center of Shahrekord Azad University. The extraction was performed by the vacuum distillation method after soaking 500 grams of the dried plant in 75% ethanol, and the resulting extract was stored in the dark at 5°C until the moment of use.

### 3.3. Phytochemical Studies of Dracocephalum kotschyi

The total phenolic and flavonoid contents (13, 14) of the extract were evaluated by the diphenyl-1-picrylhydrazyl (DPPH) assay (15), and its antioxidant properties were assessed by the iron-reducing antioxidant power (FRAP) assay (16) in a previous study (11).

### 3.4. Animals

Forty-two healthy male Wistar rats (*Rattus norvegicus*), weighing 180 ± 25 g, were obtained from the animal house of the Faculty of Veterinary Medicine, Shahrekord Azad University. The animals were maintained for one week at 22 - 25°C, with a 12-hour light/dark cycle, and had free access to tap water, along with a standard diet, to get acclimated to the laboratory environment. Female rats were not used due to the possible effects of sex hormones and the estrous cycle on liver function during the experimental period.

### 3.5. Experimental Design

The rats were randomly divided into six groups. Group 1 received 1 mL of distilled water intraperitoneally (IP) as the control. Group 2 received DIC (50 mg/kg IP). Groups 3, 4, and 5 received DIC (50 mg/kg IP), followed one hour later by the administration of HEDK (40, 80, and 120 mg/kg by gavage) (DIC + HEDK40, 80, 120). Group 6 received DIC (50 mg/kg IP), followed one hour later by the administration of SLY (100 mg/kg by gavage) (DIC + SLY). The amount of extract was chosen according to other studies on *D. kotschyi* (11, 17, 18). To look for possible side effects of HEDK, a preliminary test was conducted on rats receiving a similar dose of HEDK, and no mortality or clinically significant toxicity was observed. Treatments were performed for 7 days. Twelve hours after the administration of the last dose, the rats were anesthetized with a standard dose of ketamine + xylazine. After blood collection from the heart, the liver was removed for histopathological studies, determination of oxidative stress factors, and examination of gene expression, IL-1β, TNF-α, NOD-like receptor protein 3 (NLRP3), and high-mobility group box 1 (HMGB1) by RT-qPCR. The blood was then centrifuged at 2000 rpm for 10 minutes to obtain the serum for biochemical analysis.

### 3.6. Biochemical Analysis

The activity of the liver enzymes such as ALT, AST, and ALP was measured using the Pars Azmoun diagnostic kits (Pars Azmoun Company, Tehran, Iran) and an autoanalyzer (BT 3000, Biotecnica, Cergy Pontoise Cedex, France).

### 3.7. Assessment of Superoxide Dismutase, Catalase, Glutathione Peroxidase, and Malondialdehyde in the Liver

The assessment of antioxidant enzyme activities such as superoxide dismutase (SOD), catalase (CAT), glutathione peroxidase (GPx), and malondialdehyde (MDA) was carried out using kits from Kiazyst Company (Iran). First, a small portion of the liver was homogenized in phosphate-buffered saline (PBS), and then the amount of oxidative stress factors was measured colorimetrically according to the kit protocol.

### 3.8. Determination of Interleukin 1 Beta, Tumor Necrosis Factor α, High-mobility Group Box 1, and NOD-like Receptor Protein 3 Gene Expression

Real-time PCR was used to assess the gene expression of proinflammatory factors IL-1β, TNF-α, HMGB1, and NLRP3 in the liver. Total RNA was synthesized using a commercial BIOZOL kit (Bioer, China). The quality of each sample was assessed using a Nanodrop 2000 spectrophotometer (Thermo Fisher Scientific, Wilmington, DE). The amount of cDNA was determined using a Prime Script^™^ kit (Takara Bio Inc., Japan). The RT-qPCR was performed using SYBR^®^ Green PCR Master Mix (Qiagen, Germany) and specific primers (Table 1). The primers were purchased from Eurogentec (Seraing, Belgium). The glyceraldehyde 3-phosphate dehydrogenase (GAPDH) gene was used as a housekeeping gene.

**Table 1.** Sequences of the Primers Used for Real-time PCR

Genes	Forward	Reverse
**IL-1β**	GAAATGCCACCTTTTGACAGTG	TGGATGCTCTCATCAGGACAG
**TNF-α**	CTGGCGTGTTCATCCGTTC	GGCTCTGAGGAGTAGACGATAA
**HMGB1**	GCGCTGGCTGGAGAGTAATGT	GATTTTGGGGCGGTACTCAGA
**NLRP3**	GTGGAGATCCTAGGTTTCTCTG	CAGGATCTCATTCTCTTGGATC
**GAPDH**	GTATCGGACGCCTGGTTAC	CTTGCCGTGGGTAGAGTCAT

Abbreviations: IL-1β, interleukin 1 beta; TNF-α, tumor necrosis factor α; HMGB1, high-mobility group box 1; NLRP3, NOD-like receptor protein 3; GAPDH, glyceraldehyde 3-phosphate dehydrogenase.

### 3.9. Liver Histological Evaluation

A section of the liver was washed with normal saline and stored in 10% formalin. After sectioning and fixation, hematoxylin and eosin (H&E) staining was performed, and histopathological changes were observed.

### 3.10. Statistical Analysis

All data were presented as mean ± standard deviation (SD) and were subjected to one-way analysis of variance (ANOVA) followed by Tukey's post-hoc test using SPSS software version 26.0. A P-value of less than 0.05 was considered statistically significant. The 2^-ΔΔCT^ method was used to analyze the gene expression of IL-1β, TNF-α, NLRP3, and HMGB1.

## 4. Results

### 4.1. The Analysis of Hydroalcoholic Extract of Dracocephalum kotschyi

The phenolic and flavonoid content, as well as the antioxidant activity of HEDK, have been previously determined in a study conducted by Rezvan and Saghaei. As evident in Table 2, the results of the said study showed that HEDK not only had significant DPPH radical scavenging activity compared to BHT, but it also contained phenols and flavonoids. It also had a significant antioxidant capacity, which was confirmed by the FRAP method (11). Flavonoids are responsible for the antioxidant properties of *D. kotschyi* extract and play an important role in its biological effects. The antioxidant and anti-inflammatory effects of aqueous and alcoholic extracts of *D. kotschyi* and its isolated compounds, such as luteolin and apigenin, have been previously demonstrated (9).

**Table 2.** Phenol and Total Flavonoid Content, Diphenyl-1-picrylhydrazyl and Iron-Reducing Antioxidant Power Radical Scavenging Activity of Hydroalcoholic Extract of *Dracocephalum kotschyi* (11) ^a^

Variables	Total Phenol Content (Mg Gallic Acid/g Dry Extract)	Total Flavonoid Content (Mg Quercetin/g Dry Extract)	FRAP Content (Millimole Fe^2+^/g Dry Herb)
**Extract**	217.10 ± 9.61	53.01 ± 3.44	34.69 ± 5.19
**BHT**	-	-	1.390 ± 0.6

Abbreviation: FRAP, iron-reducing antioxidant power.

^a^ Values are expressed as mean ± standard deviation (SD).

### 4.2. The Effects of Diclofenac and Hydroalcoholic Extract of Dracocephalum kotschyi on Serum ALT, AST, and ALP

**Figure 1.**
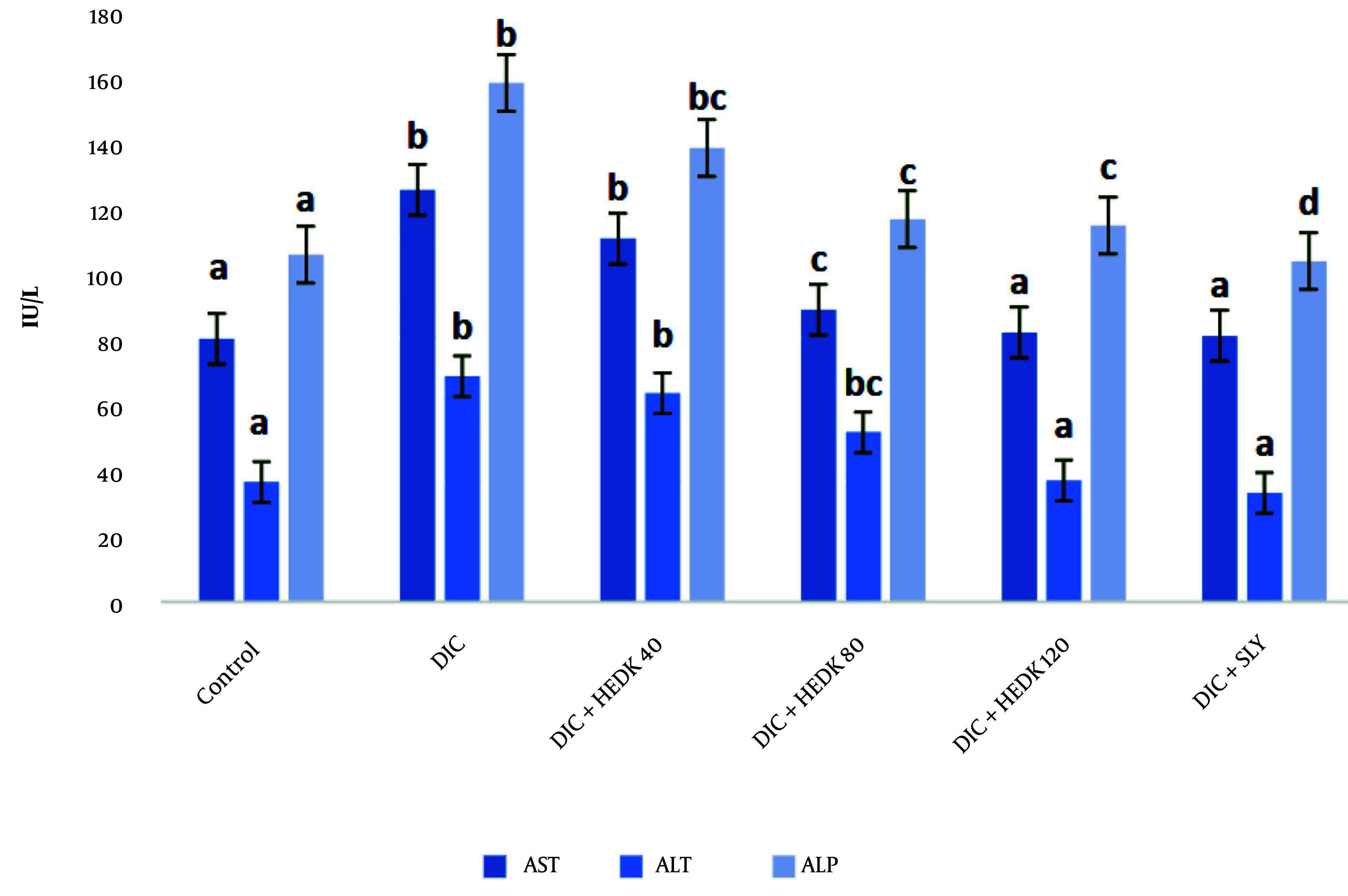
The effects of diclofenac (DIC), hydroalcoholic extract of *Dracocephalum kotschyi* (HEDK), and silymarin (SLY) on AST, ALT, and ALP in groups [the data are expressed as the means ± standard deviation (SD)]; Based on Tukey’s HSD test, groups sharing at least one common letter are not significantly different from each other (P < 0.05).

Compared to the control group, the AST, ALT, and ALP levels increased significantly after DIC administration. The HEDK reduced the target enzymes' levels dose-dependently and showed better results than SLY. The levels of these enzymes in the HEDK120 and SLY groups did not have a significant difference in comparison to the control group (Figure 1). 

### 4.3. Effects of Diclofenac and Hydroalcoholic Extract of Dracocephalum kotschyi Superoxide Dismutase, Catalase, Glutathione Peroxidase, and Malondialdehyde in the Liver

**Figure 2.**
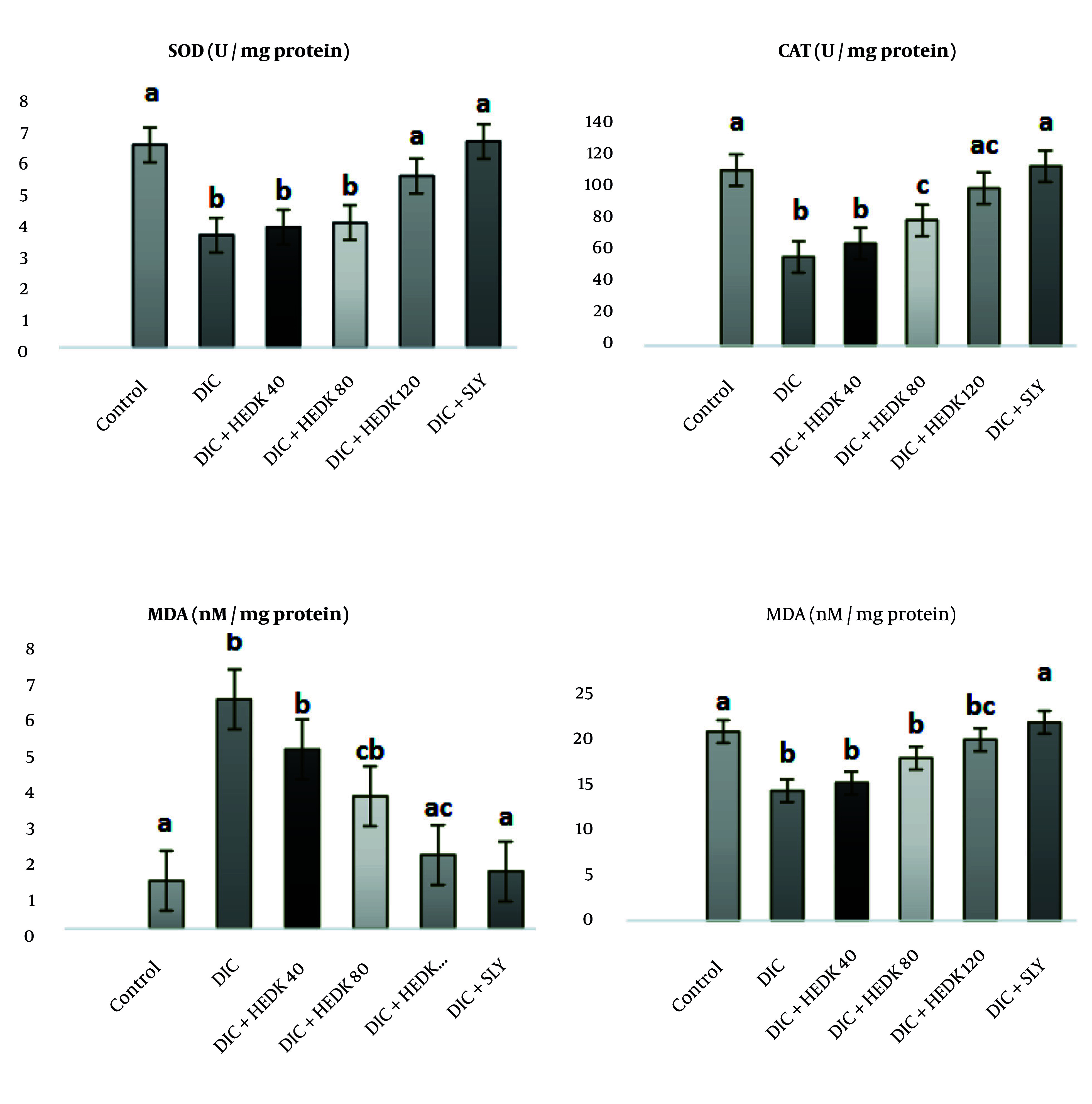
The effects of diclofenac (DIC), hydroalcoholic extract of *Dracocephalum kotschyi* (HEDK), and silymarin (SLY) on superoxide dismutase (SOD), catalase (CAT), GSH, and malondialdehyde (MDA) levels in the groups [the data are expressed as the means ± standard deviation (SD)]; Based on Tukey’s HSD test, groups sharing at least one common letter are not significantly different from each other P < 0.05).

The DIC caused a significant decrease in the activities of SOD, CAT, and GPx in the liver, along with a significant increase in the MDA level compared to the control group (P < 0.05). In contrast to the DIC group, the levels of SOD, CAT, and GPx in the liver were significantly (P < 0.05) higher after HEDK and SLY administration, whereas the MDA level in the HEDK and SLY groups had a significant drop in comparison with the DIC group (P < 0.05; Figure 2). 

### 4.4. Effects of Diclofenac, Hydroalcoholic Extract of Dracocephalum kotschyi, and Silymarin on the Expression of Interleukin 1 Beta, Tumor Necrosis Factor α, High-mobility Group Box 1, and NOD-like Receptor Protein 3 Genes in the Liver

**Figure 3.**
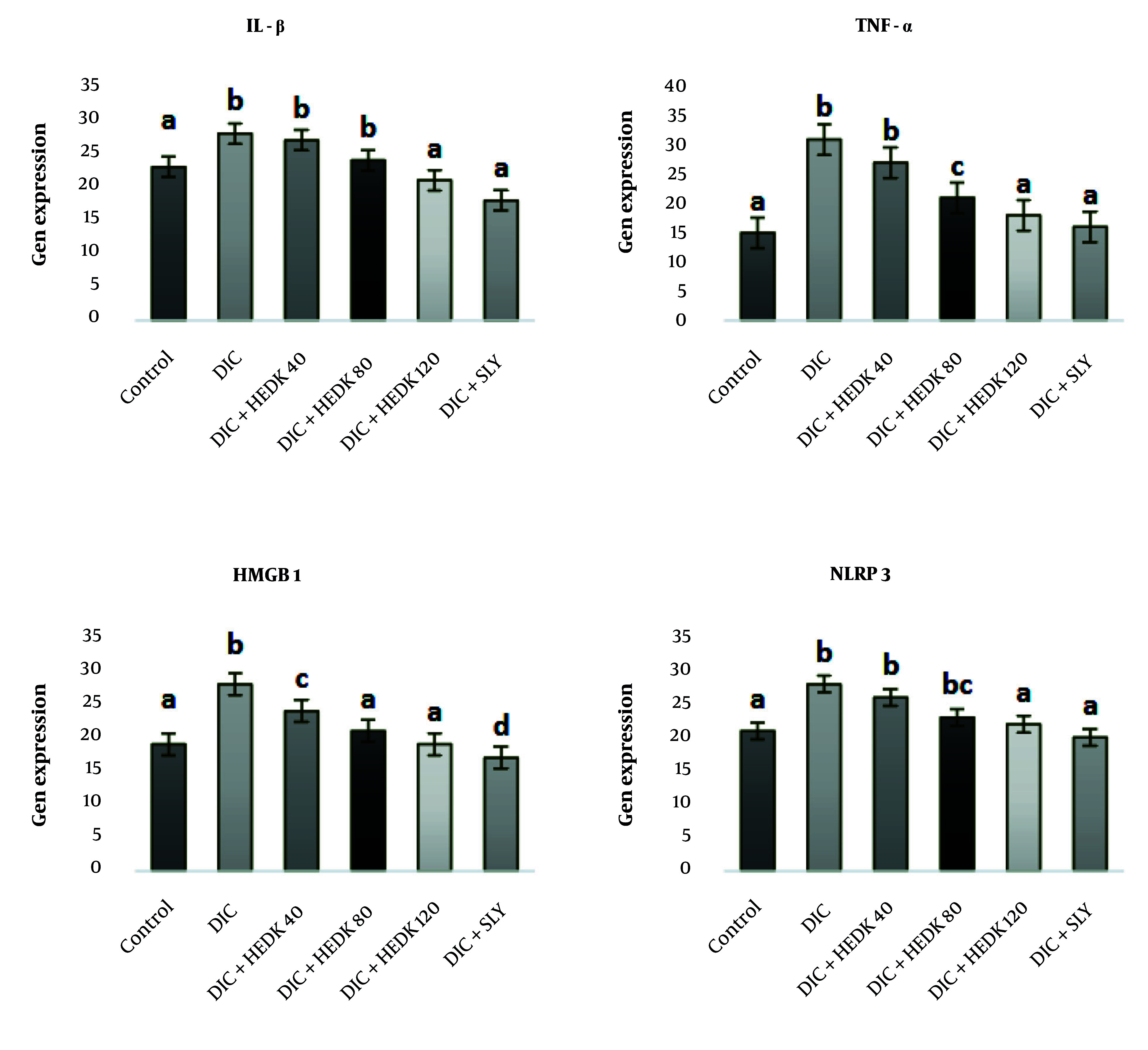
Analysis of liver proinflammatory markers; the effects of diclofenac (DIC), hydroalcoholic extract of *Dracocephalum kotschyi* (HEDK), and silymarin (SLY) on interleukin 1 beta (IL-1β), tumor necrosis factor α (TNF-α), NOD-like receptor protein 3 (NLRP3), and high-mobility group box 1 (HMGB1) gene expression [the data are expressed as the means ± standard deviation (SD)]; Based on Tukey’s HSD test, groups sharing at least one common letter are not significantly different from each other (P < 0.05).

Figure 3 shows the effects of DIC, HEDK, and SLY on the expression of IL-1β, TNF-α, HMGB1, and NLRP3 genes. The expression of IL-1β, TNF-α, HMGB1, and NLRP3 genes significantly increased after DIC administration compared to the control group (P < 0.05). In contrast, the administration of HEDK at different doses reduced the expression of these inflammatory factors. The HEDK40 reduced the expression of genes encoding IL-1β, TNF-α, NLRP3, and HMGB1, but its effect on HMGB1 was not statistically significant. The HEDK80 and HEDK120 both considerably reduced the expression of the above genes. The results of the treatment with HEDK120 were similar to those of SLY, as both reduced the expression of inflammatory genes.

### 4.5. Histopathological Findings

**Figure 4.**
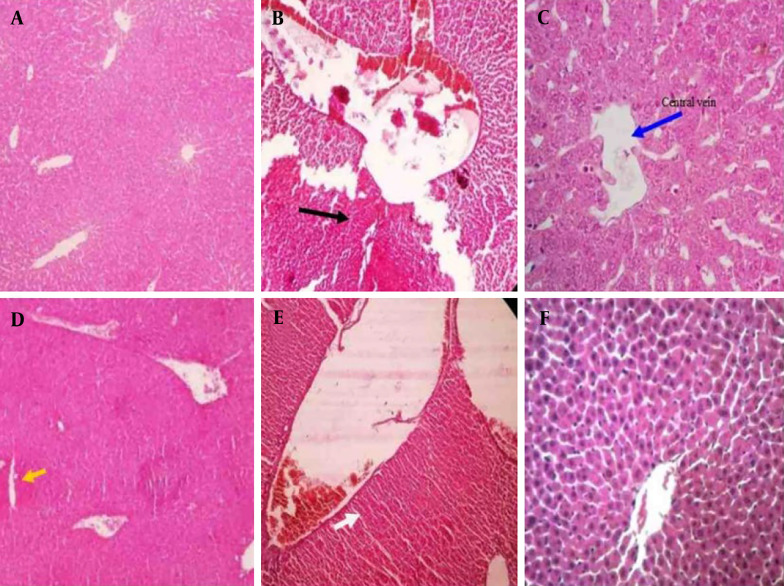
Hematoxylin and eosin (H&E) staining of the liver after different treatments in rats: A, control group with a normal structure; B, diclofenac (DIC) group (black arrow indicates necrosis); C, DIC + hydroalcoholic extract of *Dracocephalum kotschyi*40 (HEDK40) (blue arrow indicates central venous dilation); D, DIC + HEDK80 (yellow arrows indicate accumulation of edematous cells in the liver tissue and mild hepatocyte necrosis); E, DIC + HEDK120 (slight increase in the sinusoidal space); F, DIC + silymarin (SLY; with a reduction in inflammatory cell infiltration).

Figure 4 shows the histopathological results of the liver of the tested mice. As expected, a normal structure is observed in the control group (Figure 4A). However, in the DIC group, necrosis of the liver tissue and the cells around the central vein was observed, along with the dilatation of the central vein. These lesions are indicators of liver toxicity (Figure 4B). A reduction in liver damage was observed with the administration of different doses of HEDK. In the HEDK40 group, cell necrosis and dilatation of the central vein were observed (Figure 4C), and with HEDK80, mild necrosis of liver cells and accumulation of edematous cells in the liver tissue were observed (Figure 4D). The least liver damage was observed in the HEDK120 group, which included a slight increase in sinusoidal space (Figure 4E). The SLY caused a decrease in inflammatory cell infiltration compared to the DIC group (Figure 4F). 

## 5. Discussion

Elevated release of certain enzymes such as ALT, AST, and ALP into the bloodstream is often indicative of cellular damage to liver tissue (19). Reactive oxygen species cause liver damage when the body is unable to cope with oxidative stress (20). This study showed that DIC at toxic doses can cause hepatotoxicity, which is associated with increased leakage of ALT, AST, and ALP enzymes into the serum. Ezihe et al. also reported that DIC significantly increases the serum's AST and ALT levels, which is accompanied by severe liver tissue membrane damage (21). This increase in liver enzymes is reported even after topical administration of DIC (22). Various studies have confirmed DIC-induced hepatotoxicity and its prevention by some herbal compounds and natural antioxidants (23-25). GSH, an antioxidant compound, improved the function of the liver in rats receiving cyclophosphamide or suffering from renal ischemia-reperfusion injury, in addition to restoring the AST and ALP levels (20, 26).

Different doses of HEDK reduced the level of liver enzymes, with the best effect observed with HEDK120, which showed a significant difference from the enzyme levels in the DIC group and was similar to the control group. This effect of HEDK is likely related to its flavonoids and phenolic compounds, which have anti-inflammatory and antioxidant effects (9). Luteolin is one of these compounds (27) that protects the liver from acetaminophen and alcohol-induced hepatotoxicity by reducing the liver enzymes' levels (28, 29). In addition, HEDK reduced the liver’s enzyme levels in male diabetic rats. This effect was dose-dependent and statistically significant (17). The SLY, being a hepatoprotective flavonoid (23, 30), exerts its protective effects through various mechanisms, including antioxidant activities, antifibrogenic effects, and inhibition of lipid peroxidation and proinflammatory factors, in acute liver injury models (31, 32).

The HEDK and SLY both protected the liver against DIC-induced injury. The DIC and its metabolites cause hepatotoxicity through various mechanisms, such as cytochrome P450 activation, mitochondrial permeability transition, and ROS production. Balancing oxidative stress attenuates the liver cell injury induced by DIC overdose in mice (19). The lipid peroxidation product (MDA) and antioxidant enzyme activity were assessed to understand oxidative stress in liver injury. Increased ROS leads to excessive MDA production. The DIC, by affecting mitochondria and oxidative phosphorylation, and thus the production of ROS, especially O_2_, increased the MDA in the liver of rats. The significant decrease in SOD, CAT, and GPx in the liver after DIC administration indicated an increase in oxidative stress, resulting from the production of free radicals by DIC, which exacerbates hepatotoxicity through accumulation. The role of SOD in inhibiting oxidative stress involves the conversion of superoxide anions to H_2_O_2_, which is then converted to water and oxygen by GPx and CAT (21).

Administration of HEDK to DIC-exposed rats resulted in a significant increase in antioxidant enzymes (SOD, CAT, GPx), indicating the inhibitory effects of HEDK on oxidative stress. This is attributed to the phenol and flavonoids present in HEDK, which act as antioxidants. The improvement in hepatic SOD, CAT, and GPx enzyme levels with different doses of HEDK + DIC indicates that HEDK does not have oxidative properties. The inhibitory effect of HEDK on oxidative stress in acetic acid-induced colitis in rats (33) and the increase in SOD, CAT, and GPx levels, along with a decrease in MDA, in a rat model with intra-abdominal adhesions induced by abdominal surgery (11) confirm the results of the present study.

The role of HMGB1 protein in DNA replication and repair and the regulation of gene transcription is well known. Since HMGB1 is released by necrotic cells following tissue necrosis, it is considered a biomarker of the progression of inflammation and necrosis (34). In the liver, thermal shock-induced HMGB1 activates the NLRP3 inflammasome, which causes IL-1β release and inflammation, ultimately leading to liver injury (35). The HMGB1 has even been implicated in acetaminophen-induced liver injury (36). The NLRP3 inflammasome is activated in response to a variety of molecular cues, leading to the enhanced production and secretion of the proinflammatory cytokines IL-1β and TNF-α. Dysregulated NLRP3 activity leads to uncontrolled inflammation, which underlies several diseases such as gout, type 2 diabetes, atherosclerosis, and IDILI (37) and has even been identified as a key risk factor in hepatotoxicity (34). The IL-1β also plays an important role in cellular inflammation (38). The IL-1β worsens DIC-induced hepatotoxicity by upregulating immune responses (39). The TNF-α, produced by mast cells, T-cells, and macrophages, is a major factor in inducing inflammation, apoptosis, and hepatic necrosis (24).

In this study, the levels of inflammatory cytokines such as IL-1β, TNF-α, HMGB1, and NLRP3 were increased in the DIC group. This finding is consistent with the results of previous studies that a reduction in inflammation and liver fibrosis has been observed in mice after administration of an NLRP3 inflammasome inhibitor (40). In the study by Lee et al., DIC-induced hepatotoxicity in mice was accompanied by activation of immune cells and release of cytokines and chemokines, and the release of inflammatory mediators activated the TLR4/NF-κB pathway (5).

In another study, the induction of liver injury by lipopolysaccharide/DIC in mice showed a significant increase in the gene expressions of TLR4, NF-κB, IL-6, and TNF-α, and serum C-reactive protein (CRP) levels (41). In the present study, the gene expressions of IL-1β, TNF-α, HMGB1, and NLRP3 were significantly lower in the HEDK and SLY groups than in the DIC group due to the capacity of HEDK and SLY to reverse the inflammation induced by DIC toxicity. Several studies have previously demonstrated the anti-inflammatory effect of HEDK. Kalantar et al. examined HEDK about the expression of inflammatory mediators in activated macrophages. The results showed that HEDK significantly reduced the expression of inflammatory mediators such as iNOS, NF-κB, and cytokines IL-1β and TNF-α, and therefore may have beneficial effects on inflammatory diseases (42). This effect has also been confirmed in the inhibition of surgically induced intra-abdominal adhesion in rats by reducing the expression of IL-1β and TNF-α genes and the inhibition of inflammatory parameters in acetic acid-induced colitis in mice by HEDK (11, 12).

In this study, SLY treatment significantly downregulated the expression of IL-1β, TNF-α, HMGB1, and NLRP3. Previous studies have demonstrated that SLY suppresses key inflammatory mediators, including NF-κB, as well as inflammatory metabolites such as prostaglandin E2 (PGE2). Moreover, SLY exhibits potent inhibitory effects on leukotriene B4 (LTB4) (43). The role of SLY in downregulating the expression of TNF-α and IL-1β genes in drug-induced hepatotoxicity has also been reported (1, 23, 44, 45). On the other hand, SLY can ameliorate cisplatin-induced nephrotoxicity by reducing the expression of TNF-α and NF-κB genes (46). These findings are consistent with the results of the present study. In histopathological studies, hepatocellular necrosis, central vein dilation, and pericentral vein cell necrosis were observed with DIC administration compared with those in the control group, indicating hepatotoxicity. This finding was consistent with prior studies demonstrating DIC-induced hepatotoxicity in mice (1, 23, 24). Umoh et al. reported that NSAIDs, especially at high doses, may have deleterious effects on liver cell structure, which can lead to liver injury (47). In the groups that received HEDK or were treated with SLY, improvements in the liver tissue and a reduction in inflammatory cell infiltration were observed. This finding is consistent with previous studies showing that SLY prevents DIC-induced hepatotoxicity in mice (48, 49).

### 5.1. Conclusions

The administration of DIC induced hepatotoxicity, as evidenced by histopathological changes in liver tissue, elevated liver enzyme levels, upregulation of pro-inflammatory genes, and oxidative stress. In contrast, treatment with HEDK demonstrated hepatoprotective effects, significantly attenuating oxidative stress, reducing liver enzyme levels, and suppressing pro-inflammatory gene expression. These findings suggest that HEDK has promising potential to counteract DIC-induced liver toxicity.

## Data Availability

The dataset presented in the study is available on request from the corresponding author during submission or after publication.
